# RETRACTED: Serebrovska et al. Intermittent Hypoxia-Hyperoxia Training Improves Cognitive Function and Decreases Circulating Biomarkers of Alzheimer’s Disease in Patients with Mild Cognitive Impairment: A Pilot Study. *Int. J. Mol. Sci.* 2019, *20*, 5405

**DOI:** 10.3390/ijms25095039

**Published:** 2024-05-07

**Authors:** Zoya O. Serebrovska, Tetiana V. Serebrovska, Viktor A. Kholin, Lesya V. Tumanovska, Angela M. Shysh, Denis A. Pashevin, Sergii V. Goncharov, Dmytro Stroy, Oksana N. Grib, Valeriy B. Shatylo, Natalia Yu. Bachinskaya, Egor Egorov, Lei Xi, Victor E. Dosenko

**Affiliations:** 1 Department of General and Molecular Pathophysiology, Bogomoletz Institute of Physiology, 01024 Kyiv, Ukraine; belyak-serebrovska@hotmail.com (Z.O.S.); ltumanovskaya@gmail.com (L.V.T.); angela@biph.kiev.ua (A.M.S.); den-win@ukr.net (D.A.P.); goncharov@biph.kiev.ua (S.V.G.); addictive.signals@gmail.com (D.S.); dosenko@biph.kiev.ua (V.E.D.); 2 Department of Hypoxia, Bogomoletz Institute of Physiology, 01024 Kyiv, Ukraine; 3 Department of Age Physiology and Pathology of Nervous System, Chebotarev Institute of Gerontology NAMS of Ukraine, 04114 Kyiv, Ukraine; victorkholin@yahoo.com (V.A.K.); n.y.bachinskaya@gmail.com (N.Y.B.); 4 Department of Clinical Physiology and Pathology of Internal Organs, Chebotarev Institute of Gerontology NAMS of Ukraine, 04114 Kyiv, Ukraine; ksuna.m.o@ukr.net (O.N.G.); vshatilo@ukr.net (V.B.S.); 5 CellAir Constructions GmbH, 73614 Schorndorf, Germany; egorov@cellgym.de; 6 Department of Internal Medicine, Virginia Commonwealth University, Richmond, VA 23298-0204, USA

The journal *Int. J. Mol. Sci*. retracts the article “Intermittent Hypoxia-Hyperoxia Training Improves Cognitive Function and Decreases Circulating Biomarkers of Alzheimer’s Disease in Patients with Mild Cognitive Impairment: A Pilot Study” [1], cited above.

Following publication, concerns were brought to the attention of the publisher regarding a potential duplication of sections within a western blot presented within this paper [1].

Adhering to our complaints procedure, an investigation was conducted by the Editorial Office and Editorial Board that confirmed duplicated smear patterns contained with the western bots present in Figure 2. While the authors fully cooperated with the investigation, they were unable to satisfactorily explain this duplication, nor could they provide raw material to validate these findings. As a result, the Editorial Office and Editorial Board have lost confidence in the reliability of these findings and have decided to retract this article [1] as per MDPI’s retraction policy (https://www.mdpi.com/ethics#_bookmark30) and in line with the Committee on Publication Ethics retraction guidelines (https://publicationethics.org/retraction-guidelines).

This retraction was approved by the Editor-in-Chief of the journal *Int. J. Mol. Sci*.

The authors disagree with this retraction.
